# P-982. Online CME Improves Knowledge, Competence and Confidence of Infectious Disease Specialist in the Management of Patients who are Heavily Treatment Experienced

**DOI:** 10.1093/ofid/ofae631.1172

**Published:** 2025-01-29

**Authors:** Sara Thorpe, Donald Blatherwick, Maria B Uravich

**Affiliations:** Medscape Education, New York, New York; Medscape Education, New York, New York; Medscape Education, New York, New York

## Abstract

**Background:**

Heavily Treatment Experience-People Living with HIV (HTE-PLWH) encompass older individuals and perinatally infected adolescents facing challenges with medication adherence. This demographic, dealing with increased lifespans, often struggles with medication adherence, posing a significant challenge. In comparison to PLWH outside the HTE category, HTE individuals typically experience higher rates of comorbidities, AIDS-defining conditions, and elevated healthcare costs. These multifaceted factors collectively limit the available HIV therapies for HTE PLWH, making treatment decisions more complex. Addressing the challenges posed by HTE PLWH requires clinicians to stay informed about advances in antiretroviral therapy, ensuring they can provide optimal, patient-centered care.

**Methods:**

The CME intervention comprised of a 30- minute online text-based article authored by 1 faculty expert faculty. Response to 3 multiple choice, knowledge questions 1 self-efficacy, 5-point Likert scale confidence question were analyzed using a repeated pairs pre-/post-assessment study design. Pre- to post responses were compared using a McNemar’s test to assess statistical significance (P < .001 level). The activity posted on 9/27/2023; data were collected through 11/6/2023.

**Results:**

The analysis consisted of responses of infectious disease specialist (n=122) who answered all pre/post questions. Analysis demonstrated a significant improvement in knowledge and confidence related to managing HTE PLWH

• 91% relative change from 33% to 63% pre vs. post regarding knowledge of issues that can arise with the long-term use of antiretroviral therapy (P< .001)

• 60% relative change from 42% to 67% pre vs. post regarding the competence in applying best practices for managing HIV in aging patients (P< .001)

• 42% improved their confidence in their ability to manage HIV in aging patients- 23% v. 44% pre to post (P< .001)

**Conclusion:**

This study demonstrated the success of online, text-based CME on improving knowledge and confidence related to best practices in caring for people with HIV (PWH) and the subset who are heavily treatment experienced. These findings suggest the benefits of education that addresses clinicians’ individual needs across the continuum of their professional development.

**Disclosures:**

**All Authors**: No reported disclosures

